# Constructal law analysis of Cl^−^ transport in eyes aqueous humor

**DOI:** 10.1038/s41598-017-07357-8

**Published:** 2017-07-31

**Authors:** Umberto Lucia, Giulia Grisolia, Maria Rosa Astori

**Affiliations:** 1 Dipartimento Energia “ Galileo Ferraris”, Politecnico di Torino, Corso Duca degli Abruzzi 24, 10129 Torino, Italy; 2 Dipartimento di Oculistica, Azienda Ospedaliera Nazionale SS. Antonio e Biagio e Cesare Arrigo, Via Venezia 7, 15121 Alessandria, Italy

## Abstract

Progressive loss-of-vision related to any intraocular disorder is known as glaucoma. Secretion of aqueous humor is physiologically important to provide nutrients and oxygen to the avascular anterior segment and ensuring normal visual function, even if, nowadays reducing the secretory rate to lower intraocular pressure is a major strategy in treating glaucomatous patients. Understanding the mechanisms and regulation of aqueous humor formation is very important to develop possible new approaches to lower intraocular pressure,but today there isn’t any comprehensive model of the regulation of these component in forming aqueous humor. In this paper Construcal law is used to suggest how the Cl^−^ fluxes can determine the water inflow and outflow, and, consequently, how the intraocular pressure is controlled by these fluxes.

## Introduction

Progressive loss-of-vision related to any intraocular pressure (IOP) disorder is known as glaucoma: in particular, the primary open-angle glaucoma is the most common kind of this disease. Glaucoma represents the third most common cause of blindness in the developed countries^1^.

It is an optic neuropathy characterized by progressive damage in the optic nerve fibers and characteristic visual field losses, and frequently associated with an elevation of IOP^2^.

The IOP is the fluid pressure inside the eye. The fundamental cause of the increases in the IOP is blockage of the anterior drainage which generates an imbalance between the inflow and outflow of the aqueous humor.

The aqueous humor is produced by the ciliary epithelium at the rate of 120 *μ* L h^−1^ in order to provide nutritional and structural support for the components of the eye. It is drained at the anterior chamber angle. The dynamic balance between the aqueous inflow and outflow generates the IOP^2^.

At present, the basic physiological mechanism of aqueous humor formation by the ciliary epithelium and its regulation, is still not completely understood.

For a long time, it was believed that the mechanism of aqueous humor formation was a set of passive processes due only to diffusion and ultrafiltration, but, today, it is evident that the active secretion by the ciliary epithelium represent the fundamental mechanism for this process^3, 4^. Indeed, the ciliary epithelium, composed by two layers, nonpigmented (external boundary of the tissue, facing the aqueous side) and the pigmented epithelium, aligned in an apex-to-apex fashion, behaves as a selective barrier to various substances^5^. The tight junctions at the apices of the nonpigmented membrane blocks off the diffusion of various solutes and ions^2^. Consequently, the ions and metabolites follows a transcellular way from the stroma of the ciliary body in order to reach the posterior chamber. The ciliary epithelium pumps solutes and ions to the aqueous side generating concentration gradients, which drive the bulk flow of water into the eye (the aqueous inflow).

So, it is clear that the active ion transport represents a very interesting topic in order to obtain information on the mechanisms and their damages responsible of the glaucama diseases.

The theoretical difficulties related to this topic suggest us to change the viewpoint and introduce a thermodynamic approach related to the fluxes analysis, the Constructal law^6–17^. To do so, we consider that cells are adaptive biochemical engines, able to convert energy from one form to another by coupling metabolic and chemical reactions with transport processes; cells irreversibly consume free energy to maintain thermal and chemical processes and to sustain the transport of matter, energy and ions. Consequently, life results in an organisational process, in which the maximum conversion of available energy occurs. The fluxes across the cell membrane represent a fundamental quantity for any physical analysis; an irreversible biochemical thermodynamic approach can be introduced in the study of these fluxes and their biochemical effects. To develop this approach, some considerations must be introduced^18–24^:

1. The flows cause changes in entropy generation. Entropy generation is a measure of the irreversibility related to the flows across the cells membrane and an indication of the interactions between cells and environment; in fact, different cells manage energy in different ways due to their own nature, with different dissipations and consequent related different entropy generation;
2. The entropy generation of the cell-environment system is a global quantity. Consequently, by analyzing entropy we can obtain information on the behaviour of the whole cells, thus we can consider the cooperative effect of the different biochemical and bio-energetic processes inside the cells;
3. The cells reach their optimal asset by a selective process of interactions with their environment, with the consequent effect of the redistribution of energy and mass flows in their metabolic network, enabled by regulatory proteins. It occurs in the least time in order to allow the cells to adapt to environmental changes.

Physiologically, it is fundamental to identify how the transepithelial ion transport occurs in terms of transmembrane transport because it is very important in devising a correct model for aqueous humor or formation, and its regulation. This is the aim of this paper by using a Constructal law approach.

## Results

In contrast to the knowledge of the transport component, an in particular to Cl^−^ ion, in relation to water formation, there isn’t any comprehensive model of the regulation of these component in forming aqueous humor. In this paper we have obtained an analytical relation useful to suggest this explanation.

Indeed, the relation (8) allows us to link the variation in the pressure inside the anterior chamber of the eyes to the pH variation, and to the flux variation of the ions involved.

This equation, obtained by using the thermodynamic analysis of the mechanism, with particular interest in Constructal law, allows us to highlight that the net Cl^−^ flux (the difference between inflow and outflow) determine a change in the pH of biological structures spacialized in the water flows into and out of the anterior chamber of the eyes. As a consequence of this pH variation it follows a negative or positive pressure drop, which allows the water to inflow or outflow the eyes.

We have summarized the numerical results in Table 1. We can highlight that the pressure drop is usually of the order of ±2 mmHg. When something changes the Cl^−^ flux, then a change in the pressure drop occurs and a variation in the IOP occurs.

**Table 1:** Pressure drop evaluation of relation (8) on the basis of Cl^−^ fluxes across the ciliary body or cyliary epitelium as summarized in ref. 25.

Species	Net fluxes [*μ* Eq h^−1^ cm^−2^]	Pressure drop [mmHg]
Cat	2.89	±6.1
Toad	2.60	±5.4
Rabbit	2.25	±4.7
Bovine	1.03	±2.1

## Discussion

Secretion of aqueous humor is physiologically important. Aqueous flow provides nutrients and oxygen to the avascular anterior segment and sustains inflation of the globe, ensuring normal visual function in addition to other less well-defined functions, even if, nowadays reducing the secretory rate to lower intraocular pressure is a major strategy in treating glaucomatous patients^25^.

Understanding the mechanisms and regulation of aqueous humor formation is very important to develop possible new approaches to lower intraocular pressure, the current intervention well documented to slow the onset and progression of glaucomatous blindness^25^.

The nonpigmented cell represents site of solute and water efflux into the posterior aqueous chamber^26^ and the mechanisms is mediated by the Na,K-ATPase, which is localized to the basolateral membrane of nonpigmented cells^2^. Due to this active pumping by the Na,K-ATPase, the intracellular potassium concentration is high. As a consequence of the electrochemical gradient, the Cl^−^ ions leave the nonpigmented cells and pass into the posterior chamber^26^. The driving force for Cl^−^ ions exit is increased by the membrane hyperpolarization^2^. It is clear that the mechanisms and regulation of anion transport are important in understanding aqueous humor formation, but Cl^−^ is the anion of crucial importance^25^.

Na^+^/H^+^-exchanger mediates sodium entry into the pigmented cells, while Cl/HC0_3_ -exchanger is responsible for the Cl^−^ ion entry. These exchangers are independent, even if they are physiologically coupled via carbonic anhydrase^2^.

At present, many of the transport mechanisms underlying Cl^−^ secretion have been identified, but their regulation and integration are poorly understood^25^.

The Constructal law allows us to analyse the transport of ions across the boundary systems, and just this kind of approach results interesting in biomedical analysis. In this way we have able to suggest a model to explain how works the water pumping into and out of the anterior chamber of the eyes.

## Methods

The use of Constructal law allows us to describe how different ions have different effects on the use of energy by the cell for growth. The system we consider is the anterior chamber of the eye. It is an open system in which inflow and outflow of water occur. Due to local omeostasis, we consider the inflow-outflow process as isothermal. The time of the process is the least possible reaction time in order to allow the eye to adapt to any variation maintaining the right vision.

From a thermodynamic point of view a cell is a macroscopic system because it contains approximately 10^14^ molecules, with a concentration distribution related to energy and temperature, given by the following relations^18^:

1 $${c}_{N}={c}_{N\mathrm{,0}}\,{\rm{e}}{\rm{x}}{\rm{p}}\frac{N\,{e}_{N}}{{k}_{B}\,T}$$

where *c*_*N*_ is the concentrations related to the number of molecules, *e*_*N*_ is the energy per molecule, *k*_*B*_ (=1.38 × 10^−23^ J K^−1^ is the Boltzmann constant, *T* is the temperature, *c*_*N*0_ is the reference value of *c*_*N*_ at *e*_*N*_ = 0 J molecule^−1^, and *k*_*B*_ *T* ≈ 4 × 10^−21^ J molecule^−1^ for ordinary temperature^27^. Typical concentrations of the principal ions are ref. 28:

- Na^+^: 150 mM extracellular, 12 mM intracellular;
- K^+^: 4 mM extracellular, 140 mM intracellular;
- Ca^2+^: 1500 *μ* M extracellular, 0.1 *μ* M intracellular;
- Cl^−^: 120 mM extracellular, 4 mM intracellular.

Chemical reactions can occur only if the energy of the molecules is greater than the activation energy per mole e* of the reaction, so that the rate of reaction *r* per mole can be obtained through equation ((1)) by integration from e* to ∞, which holds to the Årrhenius’s law^28, 29^, for each mole and one direction reaction:

2 $$r={\rm{e}}{\rm{x}}{\rm{p}}(\frac{{e}^{\ast }}{RT})$$

The evolution of any chemical reaction at constant temperature *T* and constant pressure *p*, can be evaluated by using the differential of the Gibbs free energy *G*, by the condition *dG* < 0 (for spontaneous reaction) where^28^:

3 $$dG=V\,dp-S\,dT+\sum _{i}{\mu }_{i}d{N}_{i}$$

where *V* is the volume of the system considered, *S* is the entropy, *μ*_*i*_ represents the chemical potential of the *i*− th chemical species, and *N* stands for the number of particles which flow across the boundary of the system.

Within cells and across their micro-environment there is always an abundance of water, so atoms and molecules are often ions. Consequently, in relation to the distributions of the different ions there exist electric potential energy differences. In particular, cations (ions with positive charge) accumulate in low electric potential energy regions, while anions (ions with negative charges) present higher concentration at high values of electric potential energy regions^30^. The ion concentrations follow relations ((1)) with *e*_*N*_ = *qϕ*, where *q* = *Ze* is the ion charge, *ϕ* is the electric potential, *Z* is the chemical valence, *F* (=96,485.34 A s^−1^ mol^−1^) is the Faraday constant and, at ordinary temperature, *k*_*B*_ *T*/*e* = *RT*/*F* ≈ 25 mV, with e elementary charge (*e* = 1.602 × 10^−19^ A s). The electric potential can be evaluated by using the GoldmanHodgkinKatz equation^28, 30^:

4 $${\rm{\Delta }}{\rm{\varphi }}=\frac{R\,T}{F}{{\rm{l}}{\rm{o}}{\rm{g}}}_{10}(\frac{{P}_{{{\rm{Na}}}^{+}}\,{[{{\rm{Na}}}^{+}]}_{{\rm{out}}}+{P}_{{{\rm{K}}}^{+}}\,{[{{\rm{K}}}^{+}]}_{{\rm{out}}}+{P}_{{{\rm{Cl}}}^{-}}\,{[{{\rm{Cl}}}^{-}]}_{{\rm{out}}}}{{P}_{{{\rm{Na}}}^{+}}\,{[{{\rm{Na}}}^{+}]}_{{\rm{in}}}+{P}_{{{\rm{K}}}^{+}}\,{[{{\rm{K}}}^{+}]}_{{\rm{in}}}+{P}_{{{\rm{Cl}}}^{-}}\,{[{{\rm{Cl}}}^{-}]}_{{\rm{in}}}})$$

where *P* is the permeability of the ion, [A] means concentration of the A-ion, *R* is the ideal gas constant, *T* is the temperature, *F* is the Faraday constant, and out stand for outside, while in for inside. This last relation points out how the membrane potential can be changed by alterations in the conductance of one or more ions. The ion channels and transporters provide different permeability to distinct ions, such as Na^+^, K^+^, and Cl^−^.

Any change in the ions concentration changes both the membrane electric potential and the related pH of the cytoplasm, because the concentration of a chemical species and the pH can be obtained by the following relations^28, 30^:

5 $${c}_{out}={c}_{in}\,{\rm{e}}{\rm{x}}{\rm{p}}(\frac{{\rm{\Delta }}{\rm{\varphi }}}{R\,T})$$

and

6 $${\rm{\Delta }}{\rm{\varphi }}={\rm{\Delta }}{G}_{{H}^{+}}+2.3\frac{RT}{F}{\rm{\Delta }}{\rm{p}}{\rm{H}}$$

where *G* is the Gibbs potential, *F* is the Faradys constant, 2.3 Δ pH is the physiological concentration gradient, and H^+^ is the Hydrogen ion which is used by the cells in order to modulate the membrane electric potential by changing the H^+^ concentration.

Now, considering the eye and the previous hypotheses we can obtain the following relation:

7 $$dp=\frac{RT}{V}\,\frac{dc}{c}-\sum _{i}\frac{{\mu }_{i}}{V}\,d{N}_{i}-2.3\frac{RT}{VF}d{\rm{pH}}$$

which, considering that pH = log_10_[H^+^] and that *d* log_10_ $x=1/(x\,\mathrm{log}\,\mathrm{10)}$, becomes:

8 $$dp=\frac{RT}{V}\,({\rm{l}}{\rm{o}}{\rm{g}}10-\frac{2.3}{F})d{\rm{pH}}-\sum _{i}\frac{{\mu }_{i}}{V}\,d{N}_{i}$$

This last equation related the pressure variation with the pH variation of the eye and the flux of the involved ions.

## References

[1] Singh K (1993). Glaucoma: A global problem. Sem. Ophthalmol..

[2] To C-H, Do C-W (1998). Ion transport mechanisms in the formation of aqueous humor. HKJO.

[3] Bill A (1975). Blood circulation and fluid dynamics in the eye. Physiol. Rev..

[4] Cole DF (1966). Aqueous humour formation. Doc. Ophthalmol..

[5] Bill A (1986). The blood-aqueous barrier. Trans. Ophthalmol. Sac. UK.

[6] Bejan, A. *Shape and Structure, from Engineering to Nature* (Cambridge University Press, Cambridge, 2000).

[7] Bejan, A. & Lorente, S. *Design with Constructal Theory* (John Wiley & Sons, Hoboken, 2008).

[8] Bejan A, Lorente S (2010). The constructal law and the evolution of design in nature. Phil. Trans. B.

[9] Reis AH (2006). Constructal theory: from engineering to physics, and how flow systems develop shape and structure. Appl. Mech. Rev..

[10] Miguel AF (2011). The physics principle of the generation of flow configuration. Phys. Life Rev..

[11] Miguel AF, Bejan A (2009). The principle that generates dissimilar patterns inside aggregates of organisms. Physica A.

[12] Reis AH (2011). Design in nature, and the laws of physics. Phys. Life Rev..

[13] Bejan A (2005). The constructal law of organization in nature: Tree-shaped flows and body size. J. Exp. Biol..

[14] Bejan, A. *Advanced Engineering Thermodynamics* (John Wiley, New York, 2006).

[15] Bejan, A. & Lorente, S. *The Physics of Life: The Evolution of Everything* (St. Martin’s Press, New York, 2016).

[16] Reis AH (2014). Use and validity of principles of extremum of entropy production in the study of complex systems. Ann. Phys..

[17] Lucia U (2016). Constructal approach to bio-engineering: the ocular anterior chamber temperature. Sci. Rep..

[18] Lucia U (2012). Irreversibility in biophysical and biochemical engineering. Physica A.

[19] Lucia U (2014). Entropy generation approach to cell systems. Physica A.

[20] Lucia U (2014). Transport processes in biological systems: tumoral cells and human brain. Physica A.

[21] Lucia U (2014). Entropy generation and cell growth with comments for a thermodynamic anticancer approach. Physica A.

[22] Lucia U (2014). Thermodynamic approach to nano-properties of cell membrane. Physica A.

[23] Lucia U (2015). Bioengineering thermodynamics: an engineering science for thermodynamics of biosystems. IJoT.

[24] Lucia U (2015). Bioengineering thermodynamics of biological cells. Theor. Biol. and Med. Model..

[25] Do CW, Civan MM (2004). Basis of chloride transport in ciliary epithelium. J. Membrane Biol..

[26] Wiederholt, M., Helbig, H. & Korbmacher, C. Ion transport across the ciliary epithelium: lessons from cultured cells and proposed role of the carbonic anhydrase. In Botre, F., Gross, G. & Storey, B. T. (eds) *Carbonic anhydrase* (VCH Weinheim, New York, 1991).

[27] Lucia, U. & Grisolia, G. Constructal law analysis of ion transfer in living cells: normal and cancer behaviour. In Morega, A.-M. & Lorente, S. (eds) *Constructal Law Second Law Conference, CLC2017*, 348–369 (The Publishing House of the Romanian Academy, Bucharest, 2017).

[28] Atkins, P. & Paula, J. D. *Physical Chemistry for Life Sciences* (Oxford University Press, New York, 2006).

[29] Wolfe, J. Cellular thermodynamics: the molecular and macroscopic point of views (eLS-John Wiley & Sons, Chichester, 2015).

[30] Ashrafuzzaman, M. & Tuszynski, J. *Membrane Biophysics* (Springer, Berlin, 2013).

